# Efficacy and cerebral mechanisms of acupuncture for chronic obstructive pulmonary disease: study protocol for a multicenter, randomized controlled neuroimaging trial

**DOI:** 10.3389/fneur.2024.1363225

**Published:** 2024-06-26

**Authors:** Xugui Chen, Chan Xiong, Wei Xiao, Longyi Du, Meilu Liu, Yan Yu, Chunyu Liao, Chengshun Zhang, Yu Li, Bing Mao, Juanjuan Fu

**Affiliations:** ^1^Division of Pulmonary Medicine, Department of Internal Medicine, Institute of Integrated Traditional Chinese and Western Medicine, West China Hospital, Sichuan University, Chengdu, Sichuan, China; ^2^Department of Respiratory, No. 3 Affiliated Hospital of Chengdu University of Traditional Chinese Medicine (West District)/Chengdu Pidu District Hospital of Traditional Chinese Medicine, Chengdu, Sichuan, China; ^3^No. 3 Affiliated Hospital of Chengdu University of Traditional Chinese Medicine (West District)/Chengdu Pidu District Hospital of Traditional Chinese Medicine, Chengdu University of Traditional Chinese Medicine, Chengdu, Sichuan, China; ^4^Chengdu University of Traditional Chinese Medicine Affiliated Hospital, Chengdu, Sichuan, China

**Keywords:** dyspnea, chronic obstructive pulmonary disease, acupuncture, functional magnetic resonance imaging, randomized controlled trial, protocol

## Abstract

**Introduction:**

Although acupuncture is recommended by chronic obstructive pulmonary disease (COPD) treatment guidelines owing to its effects on dyspnea, the underlying neurobiological mechanisms of these effects remain unclear. This study aims to evaluate the efficacy of acupuncture in patients with stable COPD and explore the possible involvement of specific brain regions.

**Methods:**

This is a prospective, multicenter, single-blind, randomized controlled trial. A total of 90 participants will be recruited from three centers and will be randomly assigned in a 1:1 ratio to undergo acupuncture at acupoints on the disease-affected meridian (DAM) or non-acupoints on the non-affected meridian (NAM), in addition to routine pharmacological treatments. All participants will undergo 30 min of acupuncture three times a week for 8 weeks and will be followed up for 12 months. The primary outcome will be the severity of dyspnea, as measured using the Borg Dyspnea Scale and a visual analog scale at rest and after exercise. The secondary outcomes will include the multidimensional profile of dyspnea using Dyspnea-12, the modified Medical Research Council Dyspnea Scale, and the COPD assessment test; quality of life assessments using St George's Respiratory Questionnaire and the Hospital Anxiety and Depression Scale; and additional measurements of exacerbation frequency, pulmonary function, and the 6-min walking distance. Magnetic resonance imaging (MRI) will be performed before and after exercise to explore the potential neurobiological mechanisms of exertional dyspnea. Anxiety and depression will be measured and analyzed for their correlation with the activation of specific brain areas involved in dyspnea.

**Discussion:**

This randomized controlled trial aims to use a multidimensional evaluation of the efficacy of acupuncture in relieving dyspnea in patients with COPD in terms of emotion and quality of life and explore the neurobiological mechanisms underlying the effects of acupuncture on dyspnea from an imaging perspective. It is expected to provide strong evidence to support the use of acupuncture in relieving dyspnea in patients with COPD and those with aother diseases involving dyspnea. Additionally, it provides novel insights into the central mechanisms of acupuncture intervention and dyspnea.

**Trial registration:**

Chinese Clinical Trial Registry (https://www.chictr.org.cn/): ChiCTR2300071725.

## 1 Introduction

Chronic obstructive pulmonary disease (COPD), a heterogeneous lung condition characterized by respiratory abnormalities and chronic respiratory symptoms including dyspnea, cough, or sputum, has a global prevalence of 10.1% and is the third leading cause of death worldwide (1–3). As one of the most common symptoms experienced by patients with COPD, dyspnea refers to a subjective experience of breathing discomfort that limits the abilities of patients to perform daily activities, resulting in anxiety and depression, which ultimately impacts the patients' quality of life (4–7). It is also an independent risk factor for COPD exacerbation and is associated with increased mortality (8, 9). Pharmacological therapies recommended by the 2023 Global Initiative for Chronic Obstructive Lung Disease for dyspnea relief include bronchodilators and methylxanthines; however, these therapies fail to rectify refractory breathlessness in patients with advanced disease, which may even result in adverse events such as arrhythmia or exacerbation (10–12). Complementary therapy is required for these patients. Acupuncture has been recommended by the Global Initiative for Chronic Obstructive Lung Disease as a non-pharmacological therapy since 2021 (13). A recent meta-analysis revealed that acupuncture may improve breathlessness and quality of life in patients with advanced COPD (14). However, the effect of acupuncture is still controversial, and the potential mechanism of acupuncture employed for relieving dyspnea in patients with COPD is still unclear.

As is well known, dyspnea is a multidimensional subjective experience that is influenced by emotions such as anxiety and depression (4). An increasing number of imaging studies suggest that it may be related to brain responses. Functional neuroimaging has shown that respiratory control and perception are modulated by the corticolimbic system of the brain (4, 15). Breathlessness in healthy individuals, as induced by lower tidal volume, inhaled CO_2_, and respiratory occlusions, is related to the activation of brain regions within the anterior insula (AI), which means that various neuronal networks may respond to dyspnea perception (16–18). Compared with healthy controls, patients with COPD exhibit increased neural respiratory drive and decreased gray matter, especially in the anterior cingulate cortex (ACC). These changes are related to disease severity and anticipatory fear of dyspnea and physical activity (19–21). Activation of the medial prefrontal cortex and ACC in COPD patients correlated with the subjective dyspnea (22). The ACC may be the core brain mechanism responsible for dyspnea. Interestingly, it could also be the neural mechanism involved in acupuncture. Studies on humans and animals have revealed that acupuncture exerts multiple beneficial effects on patients with various diseases through its impact on the central nervous system, as evidenced by hemodynamic indicators in the brain (23, 24). Acupuncture can enhance brain network connectivity within the ACC, amygdala, hippocampal formation, and cerebellum, i.e., the default mode network (DMN) and sensorimotor network, to modulate emotion, memory, perception, antinociceptive activity, and sensorimotor functions (23–26). Acupuncture exhibits an additive effect in patients with depressive disorder by increasing functional connectivity in the amygdala and the ACC (27). Breathlessness is typically accompanied by anxiety; anxiety sensitivity is positively correlated with AI activity and affects the Bayesian sensory perception system associated with breathlessness (28). Therefore, acupuncture, dyspnea, and anxiety may involve similar mechanisms in the central nervous system of patients with COPD. The change in the ACC may be the neural mechanism of acupuncture for treating dyspnea in patients with COPD, with emotion also playing a role in the acupuncture treatment process for dyspnea.

**Figure 1:**
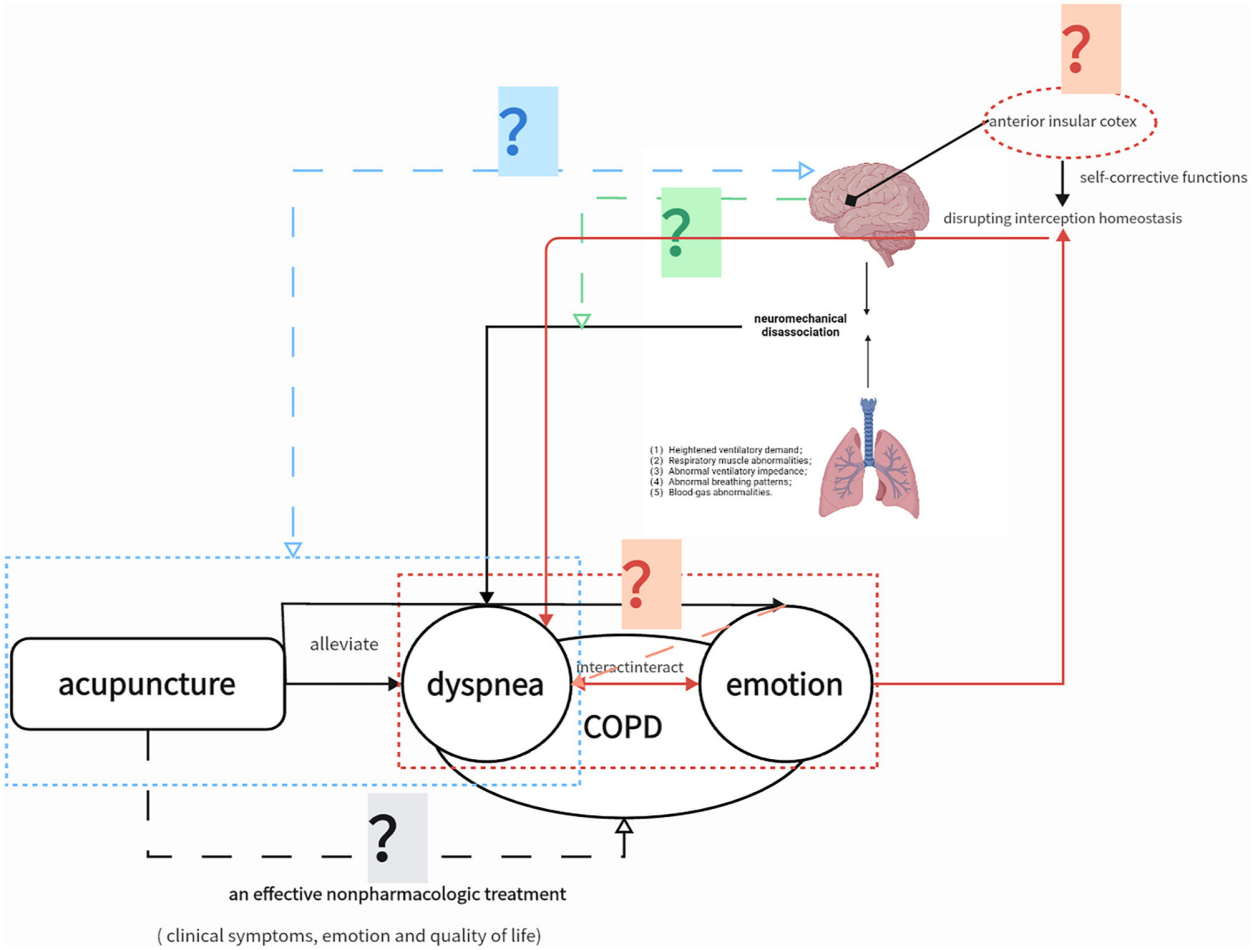
Study Hypotheses. The solid lines represent confirmed associations. The gray question mark indicates that the clinical efficacy of acupuncture in chronic obstructive pulmonary disease (COPD) still needs higher-quality clinical evidence; the blue question mark indicates that the neural mechanism of acupuncture for relieving dyspnea is still unknown; the red question mark indicates that the neural mechanism of acupuncture to relieve emotion affect is unknown, and its neural mechanism may overlap with dyspnea. The question mark indicates that the neural mechanism of exertional dyspnea induced by the 6-min walking test (6MWT) is unknown.

However, the evidence from studies on acupuncture for COPD was limited due to methodological heterogeneity, low power, and the potential morphine-sparing effects of acupuncture (14). Additionally, the mechanism underlying the effects of acupuncture on the central nervous system in COPD has not been explored. The clinical efficacy and neural mechanism of acupuncture for reducing dyspnea severity require further assessment in well-designed clinical trials. Therefore, this randomized controlled functional imaging study will examine the effects of acupuncture on the severity of dyspnea, ability to perform physical activity, and affective state in patients with COPD and explore the neurobiological mechanisms underlying the effects of acupuncture through neuroimaging. The purpose of this study is illustrated in Figure 1. We hypothesize that (1) acupuncture is an effective non-pharmacologic treatment for COPD, resulting in improvements in dyspnea, emotional state, and quality of life; (2) the alleviation of dyspnea through acupuncture is associated with abnormal structural changes and functional connections in specific brain regions; (3) acupuncture can improve long-term anxiety and depression in patients with stable COPD, with associated functional connections in specific brain regions, which may also be related to the improvement of dyspnea conditions; and (4) neural mechanisms in patients with stable COPD differ between exertional dyspnea and resting states.

## 2 Methods and analysis

### 2.1 Study design

**Figure 2:**
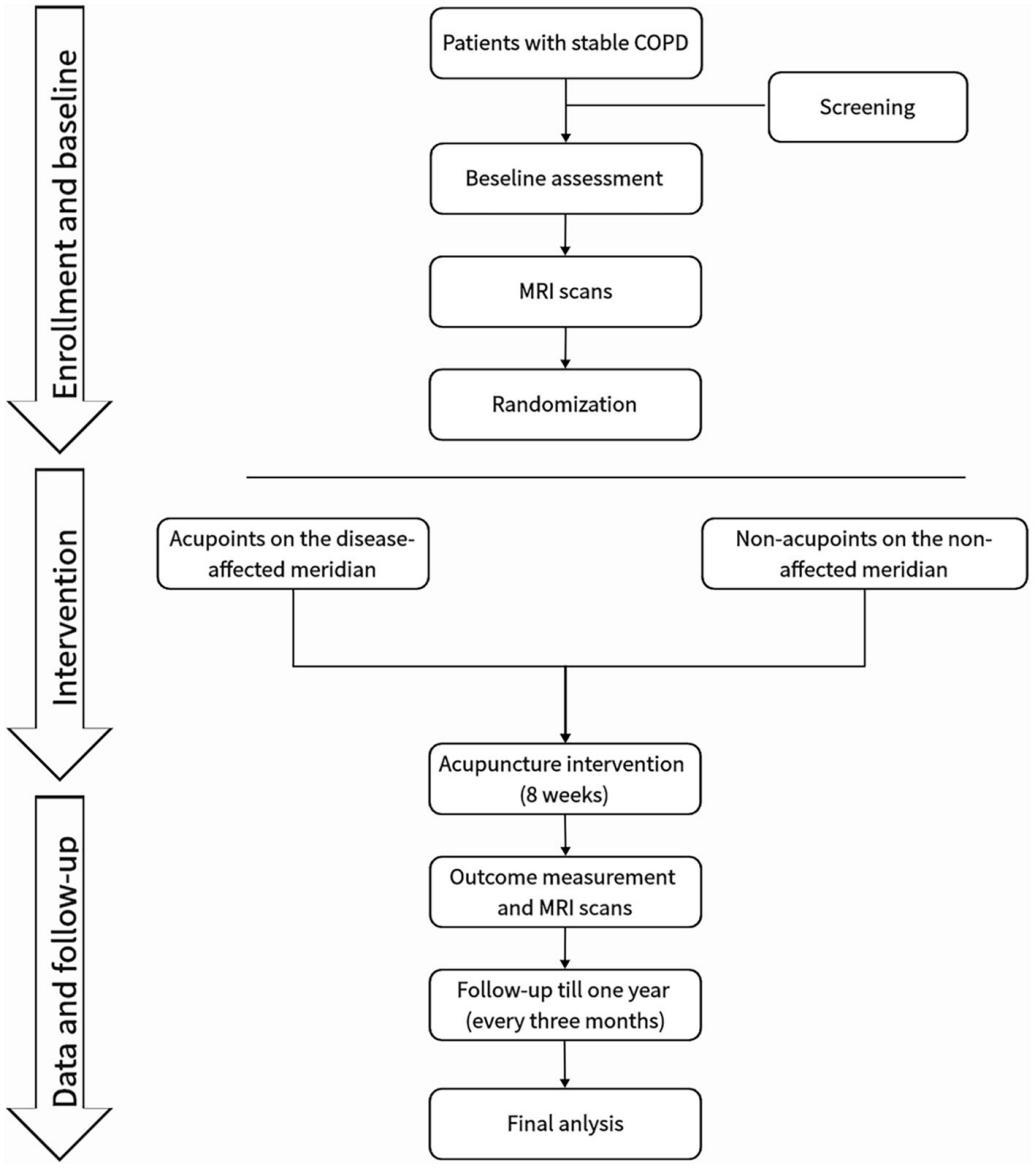
Study flowchart.

This is a prospective, multicenter, randomized, single-blind trial with two parallel groups involving patients with stable COPD. As shown in Figure 2, sociodemographic data will be collected, and participants will complete symptom questionnaires and undergo magnetic resonance imaging (MRI) before and after the 8-week intervention period. Patients will be followed up for 12 months via telephone interviews to perform COPD assessment test (CAT) and record COPD exacerbations. The exact timing of each outcome measurement is presented in Table 1.

**Table 1:** Study schedule.

**Measurements**	**Screening visit**	**Visit 1 baseline**	**Visit 2 8 weeks**	**Phone interview 6 months**	**Phone interview 9 months**	**Phone interview 12 months**
Demographics, smoking history, and medical and treatment history	×	×				
Spirometry		×	×			
Dyspnea (Borg dyspnea scale and VAS)		×	×			
Dyspnea-12		×	×			
mMRC	×	×	×			
CAT		×	×	×	×	×
SGRQ		×	×			
6MWT		×	×			
Exacerbation		×	×	×	×	×
HADS		×	×			
MRI		×	×			

VAS, visual analog scale; mMRC, Modified Medical Research Council Dyspnea Scale; CAT, chronic obstructive pulmonary disease (COPD) assessment test; SGRQ, St George's Respiratory Questionnaire; 6MWT, 6-min walking test; HADS, Hospital Anxiety and Depression Scale; MRI, magnetic resonance imaging.

### 2.2 Participants

#### 2.2.1 Eligibility and recruitment

Patients will be enrolled from the West China Hospital, Sichuan University; the No. 3 Affiliated Hospital of Chengdu University of Traditional Chinese Medicine (West District)/Chengdu Pidu District Hospital of Traditional Chinese Medicine; and the Chengdu University of Traditional Chinese Medicine Affiliated Hospital, in Chengdu, China. Patients diagnosed with COPD will be contacted via phone to assess their basic eligibility and willingness to participate. Interested individuals will be scheduled for screening interviews. Individuals who meet the eligibility criteria will be informed about the study details and will be asked to sign an informed consent form. COPD will be diagnosed in accordance with the 2023 Global Initiative for Chronic Obstructive Lung Disease report (7).

#### 2.2.2 Inclusion criteria

The inclusion criteria are as follows: (1) diagnosis of COPD with clinically stable symptoms and no antibiotic or corticosteroid use, emergency room visits, or any hospitalizations due to COPD exacerbation in the preceding 4 weeks; (2) moderate-to-very severe airflow obstruction, defined as the post-bronchodilator forced expiratory volume in 1 s being < 80% predicted; (3) aged 40–80 years without limitation of sex; (4) ability to walk unaided; (5) no pulmonary rehabilitation in the preceding 6 months; and (6) willing to cooperate with the study and provide written informed consent.

#### 2.2.3 Exclusion criteria

The exclusion criteria are as follows: (1) presence of severe comorbidities such as various organ dysfunctions, chronic kidney disease, and malignancy; (2) presence of other chronic lung diseases such as asthma and bronchiectasis; (3) requirement for mechanical ventilation or oxygen therapy for more than 16 h/day; (4) uncontrolled hypertension or diabetes; (5) neurological diseases or limited mobility that may affect communication and walking; (6) pregnant or breastfeeding; (7) claustrophobia or other contraindications to MRI; and (8) participation in other clinical trials within the last 6 months or at the same time.

#### 2.2.4 Withdrawal criteria

During the intervention period, enrolled participants will be withdrawn from the study if they (1) experience serious adverse reactions or unexpected events that prevent continuing participation; (2) experience serious complications or a deterioration in their condition requiring urgent treatment; (3) express a request to withdraw from the clinical trial; or (4) express the intent that do not want to complete the planned treatment.

### 2.3 Randomization and blinding

Participants will be assigned in a ratio of 1:1 to undergo acupuncture at acupoints on the disease-affected meridian (DAM) or at non-acupoints on the non-affected meridian (NAM). The randomization sequence will be strictly executed according to the random number table generated by a third party; the randomization list will be blinded to other researchers or patients. Acupuncturists will be informed of the grouping to allow correct needle placement.

### 2.4 Sample size

The sample size calculation was based on the dyspnea visual analog scale (VAS) score, as reported by Liu et al. (29). The test for paired means in PASS software version 15.0.5 (NCSS Statistical Software, Kaysville, UT, USA) was used to calculate the dyspnea VAS score differences in the placebo and acupuncture groups before and after acupuncture, which determined that a total of 78 patients with stable COPD should be included in the study to achieve a statistical power of 0.95 and a significance level of 5% for difference detection. Assuming a dropout rate of 15% established the necessity of a sample size of 45 participants per group. The sample size calculation based on the Borg Dyspnea Scale, as reported by Li et al. (30), showed that a total of 16 patients with stable COPD should be included in the study to achieve a statistical power of 0.97 and a significance level of 5% for difference detection. Assuming a dropout rate of 20%, a sample size of 10 participants per group is required. This study, being a multicenter, clinical, randomized controlled trial, requires a larger sample size. Therefore, a sample size of 90 participants is considered to be reasonable for this study.

### 2.5 Intervention

**Figure 3:**
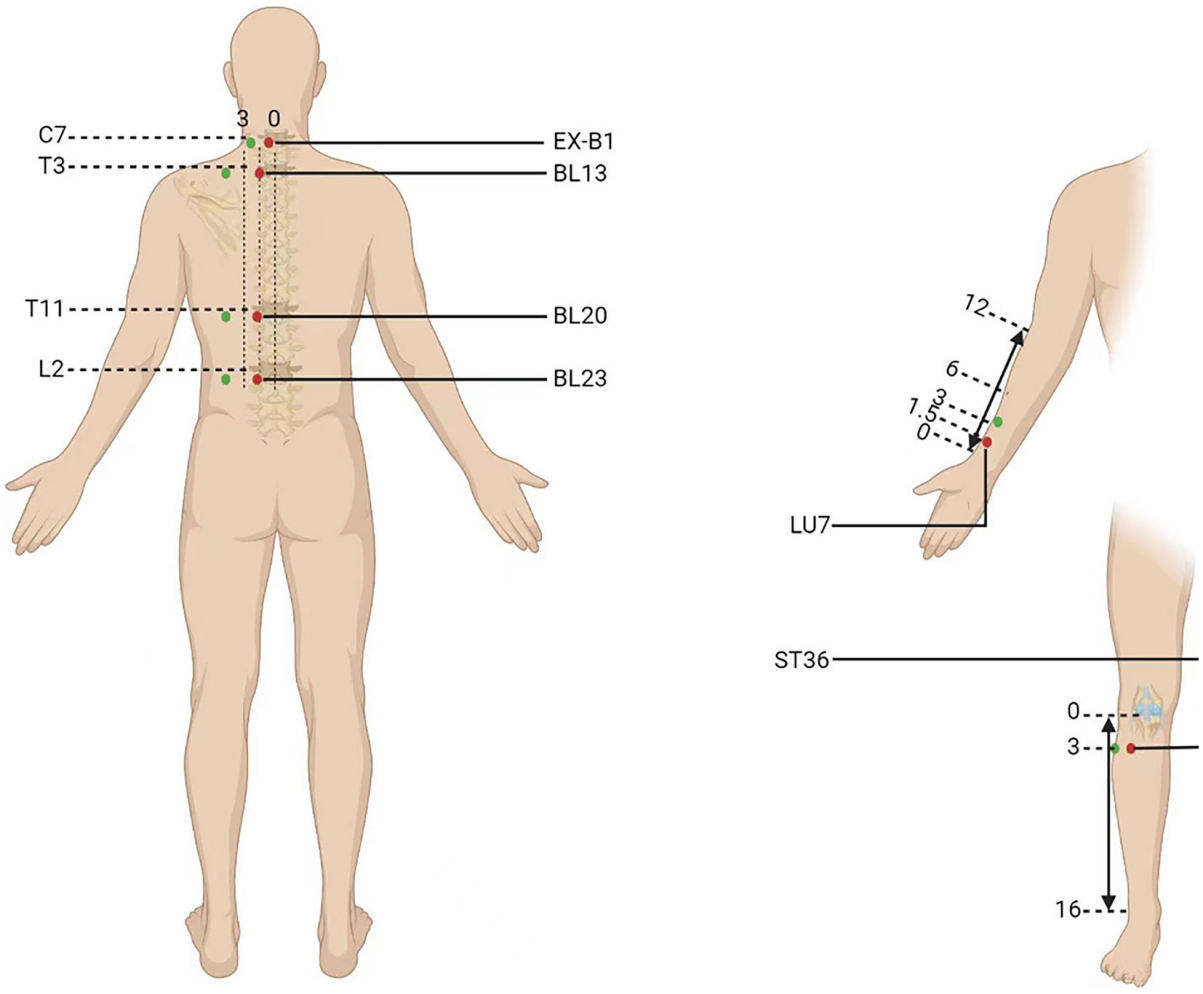
Location of acupoints or the non-acupoints on the disease-affected or non-affected meridian in the trial. The red dots are the acupoints used on the disease-affected meridian (DAM) and the green dots are the non-acupoints on the non-affected meridian (NAM).

Participants will be randomly categorized into two parallel groups to undergo acupuncture at acupoints on the DAM or non-acupoints on the NAM for 8 weeks (every 2^nd^ day, three times a week). The six standardized acupoints on the DAM are as follows: (1) EX-B1 (Dingchuan), (2) BL13 (Feishu), (3) BL20 (Pishu), (4) BL23 (Shenshu), (5) LU7 (Lieque), and (6) ST36 (Zusanli) (Table 2). The six non-acupoints on the NAM have been designed to be 2.0 cun away from the acupoints on the DAM listed above (Figure 3). Acupuncture will be performed on patients in the prone position, using disposable stainless-steel needles inserted into the disinfected skin. The locations, directions, and depths of needle insertion are presented in Table 2. Minor stimulation, achieved by lifting and thrusting the needles along with thrusting and rotating the needle sheath, will be applied to produce the sensation known as deqi (a feeling of soreness, numbness, distention, or radiation, which is considered effective). Acupuncture needles will be inserted at the intended acupoints for 30 min.

**Table 2:** Location of acupoints on the disease-affected meridian.

**Acupoints**	**Location**	**Depth**	**Direction**
Dingchuan (EX-B1)	On the back of the neck, 0.5 cun apart from the midpoint of the lower edge of the spinous process of the seventh cervical vertebra	0.5–1.0 cun	Vertically puncture or the needle point toward the spine
Feishu (BL13)	On the back, 1.5 cun apart from the spinous process of the third thoracic vertebra	0.5–0.8 cun	Oblique puncture
Pishu (BL20)	On the back, 1.5 cun apart from the spinous process of the eleventh thoracic vertebra	0.5–0.8 cun	Oblique puncture
Shenshu (BL23)	1.5 cun apart from the spinous process of the second lumbar vertebra	0.5–1.0 cun	Vertically puncture
Lieque (LU7)	On the radial side of the forearm and above the radial styloid process, between the brachioradialis muscle and the abductor pollicis longus tendon, 1.5 cun above wrist crease	0.2–0.3 cun	Upward oblique puncture
Zusanli (ST36)	On the outside of the shank, 3 cun below Dubi^*^ and a thumb lateral to the anterior tibial ridge	1.0–2.0 cun	Vertically puncture

^*^Dubi (ST35): when the knee is bent at a right angle, the acupoint is located at the concave position on the outer side of the patellar ligament of the knee joint.

### 2.6 Outcome measurement

All outcomes will be measured at baseline and after 8 weeks of intervention treatment.

#### 2.6.1 Primary outcomes

The primary outcome is the severity of dyspnea measured after the 6-min walking test (6MWT). The severity of dyspnea at rest and exertional dyspnea after 6MWT will be assessed using the Borg Dyspnea Scale and a VAS, which are convenient, simple to use, and often used for measuring exertional dyspnea; scores will be recorded within 1 min of finishing the 6MWT.

##### 2.6.1.1 Borg Dyspnea Scale

The Borg Dyspnea Scale is a modified Borg scale of perceived exertion appropriate for measuring dyspnea (31).

##### 2.6.1.2 VAS

The VAS is a line ranging from 0 (indicating no breathlessness) to 10 (indicating the most severe breathlessness imaginable), which represents the severity of dyspnea in increments of 1 cm (32).

#### 2.6.2 Secondary outcomes

##### 2.6.2.1 Modified Medical Research Council Dyspnea Scale

The modified Medical Research Council Dyspnea Scale is a 5-point (0–4) questionnaire developed to measure breathlessness; its score relates well to other multidimensional health status measures (33).

##### 2.6.2.2 Multidimensional instruments for dyspnea measurement

Dyspnea-12, a multidimensional measurement method, is composed of 12 dyspnea descriptors that can adequately reflect the physical and emotional aspects of dyspnea and its perception, offering a reliable evaluation of its severity (34).

##### 2.6.2.3 CAT

The CAT contains eight questions related to COPD symptoms, which patients answer according to their condition (35). In addition to assessing dyspnea, the test evaluates other COPD symptoms, including cough, sputum, and the ability to perform exercise.

##### 2.6.2.4 St George's Respiratory Questionnaire

The St George's Respiratory Questionnaire is the most widely documented comprehensive measure for assessing the quality of life in patients with COPD (36).

##### 2.6.2.5 Pulmonary function tests

Pulmonary function tests will be performed by professional assessors trained in spirometry (37). Participants will be asked to discontinue the use of bronchodilators before the test. Three measurements of the post-bronchodilator forced expiratory volume in 1 s and forced vital capacity will be recorded to diagnose COPD and assess the severity of airflow obstruction.

##### 2.6.2.6 6MWT

The 6MWT assesses exercise tolerance and laboratory-induced dyspnea. The test will be performed according to the guidelines issued by the American Thoracic Society in 2002 (38). After sitting at rest in a chair for at least 10 min, participants will walk back and forth to cover as much distance as possible for 6 min in a 30-m long flat and straight corridor. Rest will be allowed during the test; however, if the test ends early, the walking distance, time stopped, and the reason for stopping early will be recorded. If the test is stopped due to severe breathlessness, palpitations, or other signs of discomfort, the investigator will record the time and distance. The 6-min walk distance will be used as the outcome measure. Before and after the test, heart rate, oxygen saturation, Borg Dyspnea Scale score, and VAS score will be measured. Monitoring the heart rate will enable the assessment of exercise intensity.

##### 2.6.2.7 Hospital Anxiety and Depression Scale

The Hospital Anxiety and Depression Scale is a patient-reported score based on a 14-item questionnaire to assess the severity of anxiety and depressive symptoms and is an effective and reliable method of screening for identifying emotional disorders during hospitalization (39). Participants select the answers that best reflect their current emotional state.

### 2.7 MRI

Participants will undergo MRI four times, performed using a 3.0 T superconductor (Magnetom Skyra; Siemens Healthineers, Erlangen, Germany). At baseline, resting-state MRI will be performed after symptom assessment, and post-exercise MRI will be performed immediately after the 6MWT to capture images during dyspnea. After the intervention period, resting-state and post-exercise MRI will be repeated. While undergoing an MRI scan, the participants will be required to maintain head and neck stability and keep their eyes open. A whole-brain anatomical image consisting of 192 sagittal slices with a thickness of 1 mm will be collected using a T1-weighted 3D sequence with the following parameters: repetition time = 2,300 ms, echo time = 2.26 ms, flip angle = 8°, voxel size = 1 × 1 × 1 mm, and inversion time = 900 ms. Functional MRI will be performed using an echo-planar imaging sequence with the following parameters: repetition time = 2,000 ms, echo time = 30 ms, flip angle = 70°, voxel size = 2 × 2 × 2.5 mm, and number of slices = 54. In addition, diffusion tensor imaging will be performed using the following parameters: repetition time = 5,100 ms, echo time = 80 ms, and voxel size = 1.7 × 1.7 × 2 mm.

Two experienced radiologists will review all images to identify abnormalities including brain damage, structural brain lesions, and artifacts.

### 2.8 Statistical analysis

Qualitative variables will be expressed as numbers (percentages), and quantitative variables will be expressed as mean (standard deviation) or median (interquartile range [25%−75%], q1–q3). All efficacy evaluation data will be summarized according to the full analysis set and the per-protocol set. For safety analysis, the safety set will include all randomized participants who received at least one interventional treatment. A paired *t*-test or Wilcoxon signed-rank test will be conducted to compare the outcomes at baseline with those after the 8-week intervention period and during follow-up within a group. Analysis of covariance will be performed to compare the results after acupuncture and during follow-up between the groups. Exercise intensity will be adjusted to assess post-exercise dyspnea. Data will be analyzed using SPSS software version 26.0 (IBM Corporation, Armonk, NY, USA), and statistical significance will be set as a *p*-value of < 0.05.

Metadata from DICOM files will be converted to BIDS using HeuDiConv. fMRIPrep software version 21.0.4, a combination of tools from common software packages including FSL, ANTs, FreeSurfer, and AFNI, was developed by the laboratory of Russ Poldrack and the Stanford Center for Reproducible Neuroscience and will be used to perform minimal data preprocessing, including skull stripping, motion correction, segmentation, co-registration, and normalization. Motion artifacts will be removed automatically using independent component analysis (40). Subsequently, graph theory-based or data-driven approaches will be used to compare the differences between the DAM and NAM groups.

PANDA, a MATLAB toolbox that can be downloaded from https://www.nitrc.org/projects/panda/ and is based on MATLAB R2016a in the Ubuntu 16.04 operating system, will be used to analyze brain diffusion images. The coupling value between the functional connection matrix and the structural joint matrix will be calculated and correlated with clinical symptoms.

### 2.9 Safety control

Acupuncture-related adverse events such as fainting, pain, bleeding, and infections will be recorded in detail and addressed promptly. Serious adverse events will be recorded by the researcher and reported to the individual in charge of the study and the ethics committee within 24 h. The patients will receive treatment until full recovery.

## 3 Discussion

The recognized treatment effects of acupuncture on dyspnea in COPD are acknowledged in the COPD guidelines; however, its clinical application has been hampered by the low quality of evidence supporting its use and the lack of mechanistic studies. This randomized controlled trial aims to (1) conduct a well-designed 8-week acupuncture treatment program to observe its clinical efficacy in patients with stable COPD and to identify the location and pattern of brain activities related to dyspnea before and after treatment via functional and structural neuroimaging; (2) to explore the neural mechanisms of exertional dyspnea in patients with COPD via brain MRI using several graph theory-based or data-driven methods; and (3) to explore the comprehensive impact of acupuncture on dyspnea in multiple dimensions, particularly the affective dimension.

In this study, the acupoints commonly used for the treatment of COPD include Zusanli, Dingchuan, Lieque, Feishu, Pishu, and Shenshu (41). Acupuncture on Dingchuan can reduce the heart rate and dyspneic sensation in acute exacerbations of COPD and improve lung function in terms of forced expiratory volume in 1 s in patients with asthma (42, 43). Acupuncture on Zusanli, Pishu, Feishu, and Shenshu has been shown to improve pulmonary function and CAT scores and suppress inflammation in COPD (29, 44, 45). Acupuncture on Lieque and Zusanli has been reported to reduce nicotine cravings, and activation of the ACC, insula, and prefrontal cortex was found to be related to acupuncture (46). Stimulation of Zusanli evokes long-lasting activation of the supplementary motor cortex and deactivation of limbic/paralimbic and subcortical regions; the effect on these regions was extensive, and they were identified as network hubs related to the regulation of the secondary somatosensory cortex, emotion, and pain (23, 26, 47–49). Acupuncture on Zusanli improved the symptoms of functional dyspepsia by modulating the functional connectivity of the DMN (50). However, the target brain regions regulated by acupoints and the cumulative effects of stimulation were not determined in this randomized controlled trial on dyspnea.

Acupuncture, an important facet of traditional Chinese medicine, has demonstrated clinical efficacy in many conditions, including pain, mood disorders, and stroke, by modulating neural responses in the brain (51–53). Brain activation leads to enhanced blood flow, blood volume, and oxygen saturation in the cerebrum, thereby increasing deoxyhemoglobin levels, which is proportional to the magnetic resonance signal on functional MRI. The involvement of the sensorimotor and corticolimbic structures, which contributes to motor cognitive and emotional processing, in dyspnea perception is widely recognized (4, 25, 54). Among the brain areas related to dyspnea in COPD, the insular cortex is the most consistently reported region of sensory perception, followed by the amygdala, ACC, supplementary motor area, prefrontal cortex, and thalamus, suggesting that different neuronal networks are involved in dyspnea perception (55). According to the Embodied Prediction Interoceptive Coding and Neurovisceral Integration models, limbic cortices can generate predictions by integrating past experiences and updating through comparison with incoming sensory inputs, with the perception of breathlessness derived from the processes of interaction and comparison (56). Objective measures of disease are based on the balance between sensory inputs and predictions; factors such as effects and expectations can disrupt this balance and increase the perception of breathlessness (56). For example, pulmonary rehabilitation reduces dyspnea in patients by modulating the activity of expectation-related brain regions, such as the AI, ACC, and prefrontal cortex. Therefore, we hypothesize that acupuncture may modulate the activity of corticolimbic brain regions, such as the AI, ACC, and prefrontal cortex, to maintain the balance of predictions, thereby directly reducing dyspnea.

Anxiety or depression is experienced by 30%−40% of patients with COPD (57, 58). A self-perpetuating cycle of breathlessness and anxiety occurs in which breathlessness triggers anxiety, which in turn aggravates dyspnea (59). Fatigue and depression influence the perception of dyspnea by acting on the lateral prefrontal cortex, whereas anxiety can impair the sensitivity of resistive respiratory sensation (22, 60). In COPD patients, increased dyspnea is associated with anticipatory neural activation of the hippocampus, insula, and amygdala, which play key roles in emotional enhancement processes and are related to increased levels of anxiety and depression (61, 62). Therefore, the management of dyspnea-related anxiety and depression in patients with COPD has been investigated for its ability to attenuate the perception of dyspnea. Pulmonary rehabilitation has been reported to be the most effective treatment for dyspnea in patients with stable COPD, with a significant reduction in symptoms of depression and anxiety (63, 64). Pulmonary rehabilitation targets the brain network, including the amygdala, hippocampus, ACC, AI, and superior frontal gyrus, modulating the functions of cognitive control, symptom perception, and sensory integration; this modulation may lead to improved cognitive behavioral therapy outcomes for anxiety (65, 66). Another study reported that pulmonary rehabilitation reduced breathlessness–anxiety, which was related to lower activation of attention regulation and motor network function (67). Owing to the substantial overlap of cerebral activation regions and neural functions between anxiety and acupuncture stimulation, we hypothesize that acupuncture may modulate the activity of the lateral prefrontal cortex, hippocampus, ACC, or other brain regions to alleviate dyspnea-specific anxiety and depression and indirectly reduce breathlessness in COPD patients.

Exertional dyspnea is a major symptom of COPD and may be caused by dynamic hyperinflation during exercise (68). The Borg Dyspnea Scale, a measuring tool used during the 6MWT, can identify dynamic hyperinflation in patients with moderate-to-severe COPD with high reliability (69, 70). Exertional dyspnea during the 6MWT may be caused by a mismatch between central drive and mechanical response (71), and it has also been shown to be associated with activation of the prefrontal cortex in COPD (72). A study on cortical hemodynamics in patients with COPD suggested a stronger correlation between the premotor cortex and exertional dyspnea (73). Similar results have been observed in studies on dyspnea in healthy individuals (74). Therefore, in this study, an MRI will be performed after the 6MWT to explore the mechanisms of exertional dyspnea in the brain.

Depression is considered to be associated with more severe breathlessness, and anxiety plays a crucial role in the interplay between dyspnea and depression (75, 76). The unique connection between dyspnea and acute anxiety is considered to depend largely on subjective feelings (77), which are influenced not only by exteroception but also by interception. Interceptive awareness is a set of reciprocal afferent/efferent circuits that can integrate incoming information and engage in body signal regulation, with perceptual accuracy as a component (78). Anxiety sensitivity may reduce the stability of the Bayesian sensory perception system and increase misunderstanding of symptoms and dyspnea variability (28). Different levels of anxiety indicate different AI activities (79). A recent study suggested that AI and the pre-supplementary motor area are potential candidate brain regions for performing self-corrective functions (80). Thus, we assume that AI is key to the impact of anxiety on dyspnea and will explore the functional connections between AI and other brain regions related to exertional dyspnea for explaining the potential mechanism underlying the effects of anxiety on exertional dyspnea in patients with stable COPD.

To the best of our knowledge, this study is the first to investigate the neurobiological mechanisms of acupuncture for the treatment of dyspnea in patients with COPD. Importantly, non-acupoints on the NAM have been included as a placebo control. In this study, various dyspnea assessment scales were used before and after exercise and acupuncture. Additionally, diffusion tensor imaging and functional MRI data were collected to investigate the mechanisms of exertional dyspnea and its emotional impact on patients with stable COPD. Since dyspnea is a complex experience that can be caused or influenced by many factors and dimensions, we evaluated the effects of acupuncture on dyspnea from multiple aspects, including emotions and quality of life.

### 3.1 Limitations

This study has some limitations. The use of the 6MWT for exercise may induce different levels of exercise intensity in different patients, potentially resulting in varied levels of dyspnea. We compared exercise intensity and adjusted for the outcome of dyspnea, if needed. Furthermore, dyspnea in the ventral, frontal, and medial temporal regions could induce susceptibility artifacts on MRI, which may affect the results (81, 82). Therefore, we performed independent component analysis to automatically remove motion-induced artifacts.

## 4 Conclusion

This article introduces the design and scheme of a clinical trial to study the effects and neurobiological mechanisms of acupuncture treatment of dyspnea in patients with COPD. The results of this innovative research are expected to answer the following questions: As an alternative therapy, can acupuncture effectively alleviate dyspnea in COPD? How can functional and structural imaging explain the occurrence of exertional dyspnea in COPD? Does acupuncture relieve dyspnea by regulating the respiratory-related areas of the brain? Does acupuncture relieve dyspnea by regulating areas related to emotions? The results of this trial will provide novel insights into the mechanisms of dyspnea and provide evidence and principles for the use of acupuncture to alleviate dyspnea in COPD and other diseases involving dyspnea.

## Ethics statement

The studies involving humans were approved by the Clinical Trial and Biomedical Ethics Committee of West China Hospital of Sichuan University, Medical Ethics Committee of Pidu District Hospital of Traditional Chinese Medicine, and Medical Ethics Committee of Chengdu University of Traditional Chinese Medicine Affiliated Hospital. The studies were conducted in accordance with the local legislation and institutional requirements. The participants provided their written informed consent to participate in this study.

## Author contributions

XC: Writing – review & editing, Writing – original draft, Methodology, Data curation. CX: Writing – review & editing, Writing – original draft, Resources, Project administration. LD: Writing – review & editing, Writing – original draft, Methodology. WX: Writing – review & editing, Writing – original draft, Methodology. ML: Writing – review & editing, Writing – original draft. YY: Writing – review & editing, Writing – original draft. CL: Writing – review & editing, Writing – original draft. CZ: Writing – review & editing, Writing – original draft. BM: Writing – review & editing, Writing – original draft. YL: Resources, Writing – review & editing, Writing – original draft. JF: Writing – review & editing, Writing – original draft, Supervision, Methodology, Funding acquisition.
